# Functional vulnerability and all-cause mortality among community-dwelling United States adults with a history of hepatitis or chronic liver disease

**DOI:** 10.1016/j.iliver.2026.100259

**Published:** 2026-08-24

**Authors:** Chukwuemeka E. Ogbu, Ifeanyi Momodu, Philip N. Okafor

**Affiliations:** a Department of Internal Medicine, Cape Fear Valley Health, Fayetteville, NC, 28304, USA; b Division of Gastroenterology and Hepatology, Mayo Clinic, Jacksonville, FL, 32224, USA

**Keywords:** Chronic liver disease, Hepatitis, Frailty, Functional status, Disability, National health interview survey, Mortality

## Abstract

**Background and aims:**

Frailty predicts death in advanced cirrhosis and among transplant candidates, but whether a frailty-like functional index identifies mortality risk in the far larger population of community-dwelling adults with liver disease, who are rarely assessed for it, is unsettled. We examined whether a survey-based functional vulnerability index (FVI) was associated with all-cause mortality among US adults reporting a history of hepatitis or a chronic liver condition, independent of poverty and insurance status.

**Methods:**

Retrospective cohort study using the National Health Interview Survey (2008–2018) linked to the National Death Index through December 31, 2019. Adults 18 years or older reporting a history of hepatitis or a chronic liver condition, eligible for mortality linkage, and without baseline liver cancer were included. The FVI was defined as the proportion of 9 self-reported deficits present (fair/poor self-rated health, ADL or IADL difficulty, work limitation; and difficulty walking, climbing, standing, carrying, or grasping), modeled per 0.10-unit higher score and by deficit count (0, 1, 2, or ≥ 3). Survey-weighted Cox models estimated hazard ratios (HRs) for all-cause mortality, and survey-weighted modified Poisson models estimated 5-year mortality risk ratios.

**Results:**

Among 9291 adults (1151 deaths; 59,732 person-years; mean follow-up of 6.4 years), 64.6% had no functional deficit. In the fully adjusted model, each 0.10-unit higher FVI was associated with higher mortality (HR: 1.19, 95% confidence interval [CI]: 1.15–1.22), corresponding to an HR of 1.56 (95% CI: 1.44–1.68) per 1 − SD higher FVI. Versus no deficit, mortality increased with increasing burden: 1 deficit (HR: 1.37, 95% CI: 1.05–1.80); 2 deficits (HR: 2.49, 95% CI: 1.86–3.33); and ≥3 deficits (HR: 3.29, 95% CI: 2.65–4.08). Weighted 5-year mortality increased from 3.2% with no deficit to 23.0% with ≥3 deficits. The association was nonproportional and strongest during the first 2 years (HR: 1.28, 95% CI: 1.22–1.35), but persisted beyond 5 years (HR: 1.12, 95% CI: 1.07–1.18). Multiple imputation yielded similar results (HR: 1.20, 95% CI: 1.17–1.23). Poverty predicted mortality before but not after FVI adjustment; uninsurance did not.

**Conclusion:**

Among community-dwelling US adults with hepatitis or chronic liver disease, a survey-based functional index was strongly and gradually associated with all-cause mortality, capturing risk not explained by poverty or insurance status. Brief functional assessment may identify high-risk patients well outside the transplant clinic.

## 1 Introduction

Chronic liver disease is a major and shifting cause of death in the United States (US) and worldwide. National vital statistics place chronic liver disease and cirrhosis among the leading causes of death, accounting for tens of thousands of deaths each year.^1^ Globally, liver disease is responsible for roughly two million deaths annually and about 4% of all deaths.^2^ US mortality from cirrhosis and liver cancer rose steeply over the past two decades, with the sharpest increases among younger adults,^3^ and conventional death-certificate data probably understate the true liver-related toll.^4^

The composition of liver disease has also changed. Viral hepatitis remains clinically important, but alcohol-associated disease and metabolic dysfunction-associated steatotic liver disease have become central to the burden of advanced fibrosis and cirrhosis^5^, ^6^, ^7^. Effective hepatitis C therapy has lowered mortality among treated patients,^8^ even as population aging and cardiometabolic disease add complexity to the patients seen across primary care, gastroenterology, and hepatology.

Outcomes in liver disease are shaped by the conditions in which patients live, not by hepatic injury alone. Disparities by race and ethnicity, socioeconomic position, insurance coverage, and access to specialty care are well documented.^9^^,^^10^ Adults with liver disease encounter affordability barriers and delays in care that themselves predict death,^11^ and they carry a heavier burden of healthcare barriers than adults with many other chronic conditions.^12^ Income and coverage, however, are incomplete markers of vulnerability. Two patients with similar resources can differ profoundly in mobility, independence, work capacity, and physiologic reserve, and a measure that captures these downstream consequences may add information that social indicators alone cannot.

Frailty offers a framework for that broader vulnerability. The phenotype described by Fried and colleagues framed frailty as diminished physiologic reserve and heightened susceptibility to stressors,^13^ whereas the deficit-accumulation model defines frailty as the proportion of health deficits present across multiple domains.^14^^,^^15^ Because the deficit model does not require performance testing, it can be operationalized from observable items collected in surveys or routine encounters.^16^ In community populations, deficit-based indices strongly predict long-term mortality^17^, ^18^, ^19^. Several of their components are independently prognostic, including poor self-rated health,^20^^,^^21^ dependence in activities and instrumental activities of daily living,^22^^,^^23^ and the broader constructs of disability and functional decline.^24^^,^^25^

In hepatology, frailty has moved from a peripheral idea to a central prognostic construct, chiefly in cirrhosis and transplantation. Frailty predicts waitlist mortality independent of liver disease severity,^26^ and the liver frailty index (LFI) standardized bedside assessment and improved prediction beyond clinician judgment.^27^^,^^28^ Professional guidance now treats malnutrition, sarcopenia, and frailty as core features of cirrhosis care,^29^ reflecting shared pathways of muscle depletion and physiologic decline^30^, ^31^, ^32^. Disability and caregiving burden are common outside the transplant setting,^33^^,^^34^ and functional decline before transplantation forecasts worse waitlist and posttransplant outcomes^35^, ^36^, ^37^, ^38^. Frailty is detectable earlier as well: it tracks with cirrhosis progression and death,^39^ is present in noncirrhotic fatty liver disease,^40^ and, when self-reported, predicts mortality in hospitalized cirrhosis.^41^^,^^42^

Most of this evidence comes from advanced cirrhosis, transplant evaluation, or specialty clinics. The far larger population of community-dwelling adults living with a history of hepatitis or a chronic liver condition is underrepresented; many are managed outside hepatology, may never undergo formal frailty testing, and may develop limitations in daily activities long before decompensation or referral. A community lens asks a different question: whether frailty-like deficits visible in ordinary life identify mortality risk in a nationally representative outpatient population. The National Health Interview Survey (NHIS), linked to the National Death Index, provides large, nationally representative cohorts with information on diagnosed conditions, functional limitations, and social factors, and reproduces national mortality experience well^43^, ^44^, ^45^. We evaluated whether a survey-based Functional Vulnerability Index (FVI) was associated with all-cause mortality among US adults with a history of hepatitis or chronic liver disease, and whether that association persisted beyond poverty and insurance status.

## 2 Methods

### 2.1 Design and data source

This retrospective cohort study used pooled NHIS data for 2008 through 2018 obtained through IPUMS NHIS, linked to mortality follow-up through December 31, 2019.^43^^,^^44^ NHIS is a nationally representative household survey of the civilian, non-institutionalized US population. Survey years 2008 through 2018 were used because they contained the liver disease items, functional-limitation items, complex-survey design variables, and linked mortality variables required for the analysis. Because the public-use linked mortality files are most dependable for vital status, all-cause mortality was prespecified as the primary outcome.^43^ The study used deidentified public-use data, involved no participant contact, and is reported in accordance with the Strengthening the Reporting of Observational Studies in Epidemiology guideline.^46^

### 2.2 Study population

The analysis was restricted to sample adults 18 years or older, for whom the functional-limitation items and the sample-adult mortality weight were designed. Eligible participants were those who reported a history of hepatitis or a chronic liver condition, were eligible for linked mortality follow-up, and had no baseline report of liver cancer; adults with baseline liver cancer were excluded to limit the possibility that malignancy rather than underlying liver disease or functional vulnerability drove mortality. For secondary analyses, a 3-level subgroup was defined: hepatitis only, chronic liver condition only, and both.

### 2.3 Exposure: functional vulnerability index

The FVI was constructed as a deficit-accumulation index from 9 self-reported NHIS items^16^: fair or poor self-rated health; need for help with activities of daily living; need for help with instrumental activities of daily living; limitation in the kind or amount of work, or inability to work; and at least moderate difficulty walking, climbing, standing, carrying, or grasping. Each item was scored as a deficit (1) or not (0); the FVI was the number of deficits divided by the number of non-missing items, requiring at least 7 of 9 items, and ranged from 0 to 1, with higher values indicating greater vulnerability.

Because the distribution carried a large mass at 0, the continuous FVI was modeled per 0.10-unit higher score as the primary parameterization, with the per 1 − SD estimate reported for continuity with the prior frailty literature. A categorical exposure based on the observed deficit count (0, 1, 2, or ≥ 3) was used in place of quantiles, which split the zero mass arbitrarily and can produce uninterpretable contrasts. To characterize the index rather than to validate it against the LFI, we computed item prevalence and missingness, internal consistency (Kuder-Richardson 20, equivalent to coefficient alpha for binary items), corrected item-total correlations, tetrachoric correlations with a principal-component summary, and known-groups associations with age, poverty, comorbidity burden, and liver subgroup. These psychometric statistics were interpreted descriptively because the FVI is a formative deficit-accumulation measure rather than a reflective scale.^15^^,^^16^

### 2.4 Outcome and follow-up

The outcome was all-cause mortality. Participants who died during follow-up were classified as deaths; survivors were censored at the end of 2019. Because exact interview dates were unavailable in the analytic extract, follow-up time was approximated from the survey year together with the death year and quarter; this approximation was the same for all participants and was examined directly in landmark analyses.

### 2.5 Covariates

Covariates were age, sex, race and ethnicity, educational attainment, US Census region, survey year, poverty status (below *vs*. not below the federal threshold), current insurance status (uninsured *vs.* insured), smoking status, alcohol use, body mass index, diabetes, hypertension, prior myocardial infarction, and any cancer history.

### 2.6 Statistical analysis

All analyses incorporated the NHIS complex design using strata, primary sampling units, and the sample-adult mortality weight divided by the number of pooled survey years.^47^ Survey-weighted Cox proportional hazards models estimated hazard ratios (HRs) and 95% CIs.^48^ Models were fitted sequentially: unadjusted; demographic (age, sex, race and ethnicity, education, region, survey year); plus poverty and insurance; plus health behaviors and cardio-metabolic comorbidity; and, as the primary model, plus the liver subgroup. The proportional-hazards assumption was assessed with scaled Schoenfeld residuals and a test of nonzero slope against transformed time, supplemented by a survey-weighted interaction between FVI and log follow-up time^49^; when non proportionality was detected, time-specific HRs were estimated in prespecified intervals (0–2, > 2–5, and > 5 years). Five-year mortality risk ratios were estimated with survey-weighted modified Poisson regression using a log link and a sandwich variance estimator.^50^

Because the complete-case model excluded participants with missing covariates, missingness was tabulated, and the included and excluded groups were compared, and the primary model was re-estimated under multiple imputation by chained equations (20 imputations) with the cumulative hazard and the event indicator included in the imputation model, and estimates pooled by the Rubin rules^51^, ^52^, ^53^, ^54^. To address possible reverse causation, landmark analyses excluded participants who died or were censored within the first 1 and 2 years. Prespecified sensitivity analyses varied the FVI construction (counting a non-activity variable as a deficit; counting any difficulty as a deficit; excluding self-rated health, work limitation, or both; and leaving out each item in turn), the non missing-item threshold (≥8 and 9 of 9), the age adjustment (a 4-knot spline), and the cohort definition (chronic liver condition only; an expanded definition including a recent liver condition). Effect modification by age, sex, race and ethnicity, and liver subgroup was tested with multiplicative interaction terms. Analyses used R with the survey, survival, splines, and mice packages; 2-sided *p* < 0.05 defined significance.

## 3 Results

### 3.1 Sample and follow-up

**Fig. 1:**
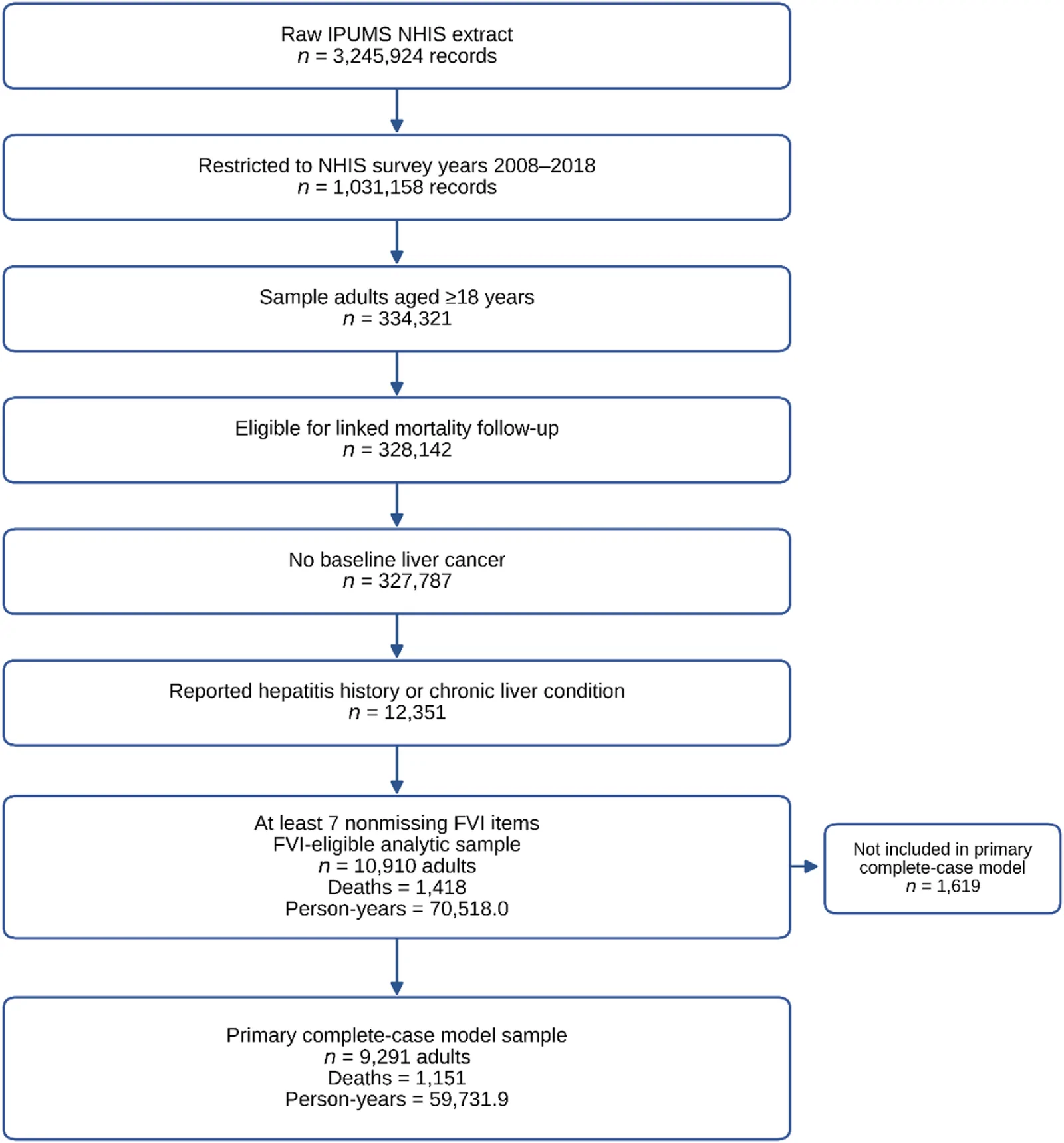
Construction of the analytic cohort. Flow of participants from the pooled 2008–2018 National Health Interview Survey extract through restriction to the survey years, sample adults 18 years or older, eligibility for linked mortality follow-up, exclusion of baseline liver cancer, restriction to a reported history of hepatitis or chronic liver condition, availability of the Functional Vulnerability Index, and the final complete-case model sample, with the number remaining and the number excluded at each step. Abbreviations: FVI, Functional Vulnerability Index.

Of the adults reporting a history of hepatitis or a chronic liver condition, 10,910 met the FVI item threshold (1418 deaths; 70,518 person-years). The primary complete-case model included 9291 adults and 1151 deaths across 59,732 person-years (mean follow-up, 6.4 years per participant) (Fig. 1, Table S1).

### 3.2 Distribution and baseline characteristics

The FVI distribution was concentrated at 0: 64.6% of adults had no functional deficit, 12.4% had 1, 5.4% had 2, and 17.5% had 3 or more (Table 1). Functional burden tracked with age (mean, 50.4 years with no deficit *vs.* 60.5 years with ≥ 3 deficits), body mass index, poverty (8.3% *vs.* 32.7%), diabetes (8.8% *vs.* 35.5%), hypertension (31.3% *vs.* 68.7%), and chronic liver condition (25.7% *vs.* 51.6%). Adults with more deficits were more often current smokers and former drinkers and less often college-educated.

**Table 1:** Baseline characteristics of the primary complete-case sample, by observed functional deficit count.

Characteristic		Overall	0	1	2	≥ 3	*p*
Unweighted No.		9,291	5,602	1,171	577	1,941	
Weighted distribution, %		100.0	64.6	12.4	5.4	17.5	
Age, mean (SE), year		53.0 (0.2)	50.4 (0.3)	54.7 (0.6)	56.5 (0.7)	60.5 (0.4)	< 0.001
Body mass index, mean (SE)		28.0 (0.1)	27.4 (0.1)	28.6 (0.2)	28.9 (0.3)	29.7 (0.2)	< 0.001
FVI, mean (SE)		0.14 (0.00)	0.00 (0.00)	0.12 (0.00)	0.25 (0.00)	0.62 (0.01)	< 0.001
Nonmissing FVI items, mean (SE)		8.5 (0.0)	8.8 (0.0)	8.3 (0.0)	8.0 (0.0)	8.0 (0.0)	< 0.001
Below poverty threshold, No. (%)		1,826 (14.6)	605 (8.3)	283 (18.3)	173 (22.7)	765 (32.7)	< 0.001
Currently uninsured, No. (%)		1,145 (12.3)	769 (13.2)	192 (16.0)	57 (9.6)	127 (7.3)	< 0.001
Diabetes, No. (%)		1,538 (15.8)	496 (8.8)	242 (21.0)	145 (23.7)	655 (35.5)	< 0.001
Hypertension, No. (%)		4,114 (41.5)	1,836 (31.3)	609 (51.1)	337 (53.4)	1,332 (68.7)	< 0.001
Prior heart attack, No. (%)		590 (6.1)	147 (2.8)	77 (7.8)	59 (10.6)	307 (15.9)	< 0.001
Any cancer history, No. (%)		1,312 (13.3)	599 (10.0)	185 (16.2)	122 (22.3)	406 (20.5)	< 0.001
Hepatitis history, No. (%)		7,526 (81.0)	4,752 (84.2)	928 (78.5)	436 (74.6)	1,410 (73.0)	< 0.001
Chronic liver condition, No. (%)		3,119 (32.9)	1,397 (25.7)	441 (38.5)	276 (46.3)	1,005 (51.6)	< 0.001
Sex, No. (%)							< 0.001
	Male	4,614 (52.2)	2,844 (53.3)	628 (55.5)	302 (52.1)	840 (45.5)	
	Female	4,677 (47.8)	2,758 (46.7)	543 (44.5)	275 (47.9)	1,101 (54.5)	
Race and ethnicity, No. (%)							< 0.001
	Non-hispanic White	5,892 (66.5)	3,503 (65.1)	737 (65.2)	383 (71.1)	1,269 (71.4)	
	Non-hispanic Black	969 (8.0)	465 (6.7)	126 (8.2)	76 (10.0)	302 (11.6)	
	Hispanic	1,706 (17.7)	1,122 (19.1)	221 (19.4)	80 (12.4)	283 (13.0)	
	Non-hispanic Other	724 (7.8)	512 (9.1)	87 (7.2)	38 (6.5)	87 (4.0)	
Education, No. (%)							< 0.001
	< High school	1,423 (13.7)	552 (9.6)	205 (16.2)	122 (18.6)	544 (25.4)	
	High school/GED	2,286 (24.4)	1,229 (21.8)	327 (27.9)	152 (27.4)	578 (30.6)	
	Some college/AA	2,936 (31.2)	1,764 (30.9)	392 (33.2)	195 (33.3)	585 (30.4)	
	Bachelor or higher	26,46 (30.6)	2,057 (37.6)	247 (22.6)	108 (20.7)	234 (13.6)	
Region, No. (%)							0.022
	Northeast	1,536 (17.4)	944 (18.3)	193 (16.2)	93 (18.3)	306 (14.7)	
	Midwest	1,690 (18.8)	1,007 (18.7)	208 (17.4)	108 (20.2)	367 (20.2)	
	South	3,304 (36.1)	1,931 (34.9)	409 (37.4)	198 (34.3)	766 (40.0)	
	West	2761 (27.7)	1720 (28.1)	361 (29.0)	178 (27.2)	502 (25.1)	
Smoking status, No. (%)							< 0.001
	Never	4,272 (48.0)	3,019 (55.4)	458 (39.1)	201 (36.2)	594 (31.0)	
	Former	2,844 (29.9)	1,616 (27.9)	377 (32.2)	194 (32.9)	657 (34.6)	
	Current	2,175 (22.0)	967 (16.7)	336 (28.7)	182 (30.9)	690 (34.4)	
Alcohol use, No. (%)							< 0.001
	Lifetime abstainer	1,528 (16.2)	898 (16.0)	174 (15.0)	78 (13.7)	378 (18.6)	
	Former drinker	2,131 (20.7)	864 (14.2)	331 (25.5)	195 (33.2)	741 (37.7)	
	Current drinker	5,632 (63.0)	3,840 (69.8)	666 (59.4)	304 (53.1)	822 (43.7)	
Liver/hepatitis subgroup, No. (%)							< 0.001
	Hepatitis only	6,172 (67.1)	4,205 (74.3)	730 (61.5)	301 (53.7)	936 (48.4)	
	Chronic liver condition only	1,765 (19.0)	850 (15.8)	243 (21.5)	141 (25.4)	531 (27.0)	
	Both conditions	1,354 (13.9)	547 (9.9)	198 (17.0)	135 (20.9)	474 (24.6)	

Note: Counts are unweighted; percentages and means are survey-weighted, so a percentage does not equal the count divided by the column total. Columns are the observed number of functional deficits (0, 1, 2, or ≥3), used in place of quantiles because most participants had an FVI of 0. Continuous variables are survey-weighted mean (SE). *p*-values are design-based (Rao–Scott F test or survey-weighted regression). Abbreviations: FVI, Functional Vulnerability Index; SE, standard error.

### 3.3 Characterization of the index

Among adults with all 9 items observed, internal consistency was high (Kuder-Richardson 20, 0.90), corrected item-total correlations ranged from 0.44 to 0.84, and a single principal component derived from the tetrachoric correlation matrix explained 86.5% of variance, consistent with a coherent functional construct rather than several unrelated items (Table S2 and S3). Component prevalence rose monotonically across deficit-count categories (Table S4). Mean FVI increased across groups expected to differ, including older age, poverty, greater comorbidity burden, and chronic liver versus hepatitis-only disease (Table S5), and mortality rose stepwise across deficit-count categories, from 7.7 to 50.0 deaths per 1000 person-years (Table S6).

### 3.4 Functional vulnerability and all-cause mortality

**Fig. 2:**
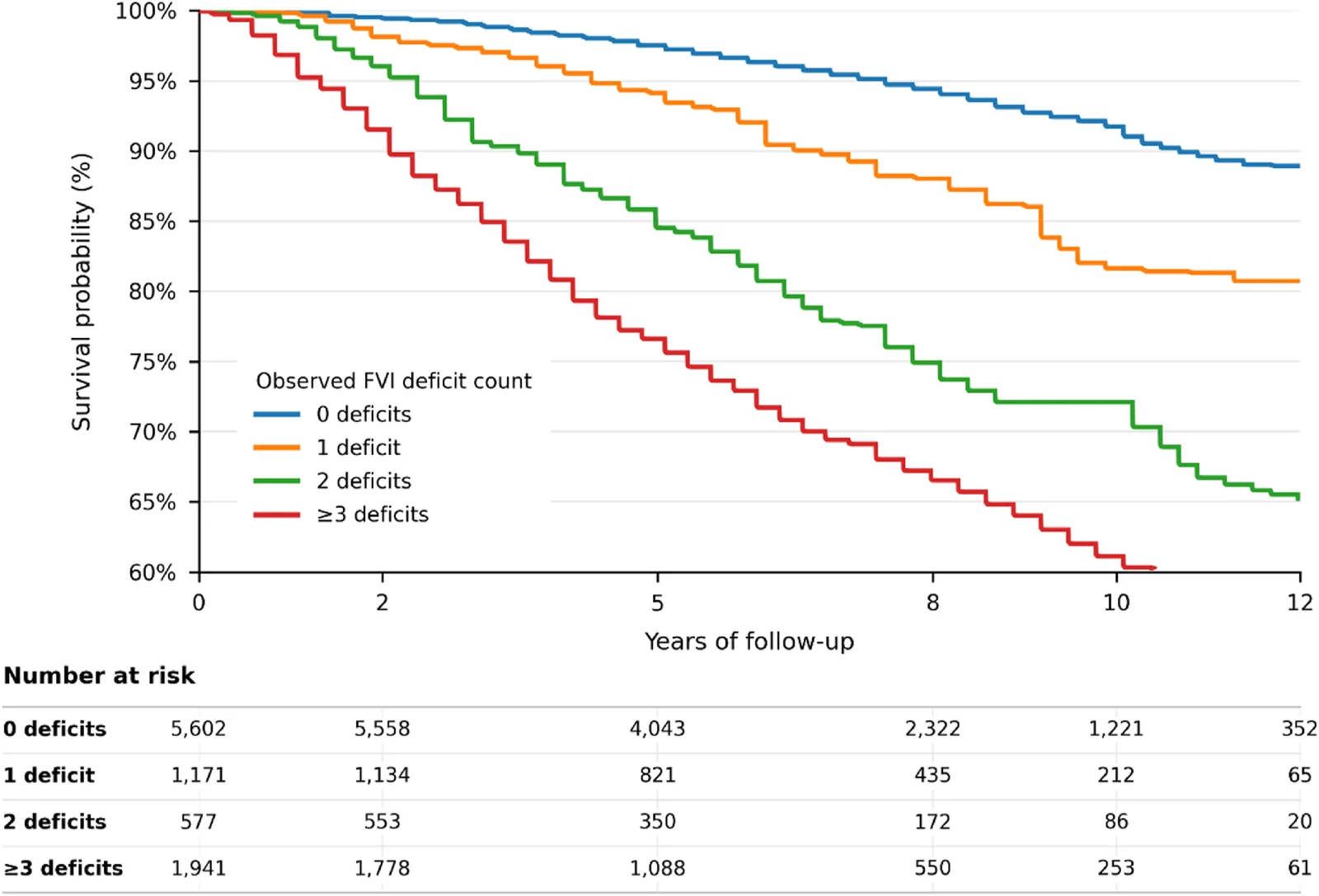
Survey-weighted survival by observed FVI deficit count. Survey-weighted Kaplan–Meier curves for all-cause mortality among participants with 0, 1, 2, or ≥3 observed deficits. The table gives the unweighted number at risk at each time point on the x-axis. Because the 2008–2018 cycles were pooled and mortality linkage ended 31 December 2019, most participants left the risk set by censoring rather than death (7642 *vs.* 1151), so these counts fall faster than the curves and, being unweighted, are not directly comparable with the weighted estimates. Note the truncated y-axis.

In the unadjusted model, each 0.10-unit higher FVI was associated with higher mortality (HR: 1.28; 95% CI: 1.25–1.31) (Table 2). The estimate was attenuated only modestly by sequential adjustment: demographics (HR: 1.23; 95% CI: 1.21–1.26), then poverty and insurance (HR: 1.23; 95% CI: 1.20–1.27), then behaviors and comorbidity (HR: 1.20; 95% CI: 1.16–1.23), and the primary model adding the liver subgroup (HR: 1.19; 95% CI: 1.15–1.22). Scaled to the SD, the fully adjusted estimate corresponded to an HR of 1.56 (95% CI: 1.44–1.68) per per 1 − SD higher FVI. Survey-weighted survival curves separated progressively across deficit-count categories (Fig. 2).

**Table 2:** Sequential survey-weighted Cox models for functional vulnerability and all-cause mortality.

Model	No.	Deaths	Person-years	HR per 0.10 higher FVI (95% CI)	*p*
Unadjusted	9,291	1,151	59,731.9	1.28 (1.25–1.31)	< 0.001
Model 1: demographic	9,291	1,151	59,731.9	1.23 (1.21–1.26)	< 0.001
Model 2: + poverty, insurance	9,291	1,151	59,731.9	1.23 (1.20–1.27)	< 0.001
Model 3: + behaviors, comorbidity	9,291	1,151	59,731.9	1.20 (1.16–1.23)	< 0.001
Model 4: + liver subgroup (primary)	9,291	1,151	59,731.9	1.19 (1.15–1.22)	< 0.001
Per 1 − SD higher FVI (fully adjusted)	9,291	1,151	59,731.9	1.56 (1.44–1.68)	< 0.001

Note: Model 1 adjusted for age, sex, race and ethnicity, education, region, and survey year. Model 2 added poverty and current insurance status. Model 3 added smoking, alcohol use, body mass index, diabetes, hypertension, prior heart attack, and cancer history. Model 4 (primary) added liver/hepatitis subgroup. The final row reports the same primary model scaled per per 1 − SD higher FVI. Abbreviations: CI, confidence interval; FVI, Functional Vulnerability Index; HR: hazard ratio; SD, standard deviation.

### 3.5 Deficit count and the nonzero range

Modeled by observed deficit count, mortality rose with burden relative to adults with no deficit (1 deficit: HR: 1.37 [95% CI: 1.05–1.80]; 2 deficits: 2.49 [1.86–3.33]; ≥ 3 deficits: 3.29 [2.65–4.08]) (Table 3). Any deficit, *vs.* none, carried an HR of 2.34 (95% CI: 1.94–2.83). Among the 3689 adults with any deficit, mortality continued to rise with the degree of vulnerability (HR per 0.10-unit, 1.15; 95% CI: 1.11–1.19), indicating a graded relationship rather than a simple threshold.

**Table 3:** Mortality by observed deficit count and within the nonzero functional vulnerability range.

Exposure contrast	No.	Deaths	HR (95% CI)	*p*
1 deficit *vs.* 0 deficits	9,291	1,151	1.37 (1.05–1.80)	0.022
2 deficits *vs.* 0 deficits	9,291	1,151	2.49 (1.86–3.33)	< 0.001
≥ 3 deficits *vs.* 0 deficits	9,291	1,151	3.29 (2.65–4.08)	< 0.001
Any deficit *vs.* none	9,291	1,151	2.34 (1.94–2.83)	< 0.001
Among FVI > 0, per 0.10 higher FVI	3,689	817	1.15 (1.11–1.19)	< 0.001

Note: All models used the fully adjusted primary covariate set, including liver/hepatitis subgroup; the reference for deficit-count contrasts is 0 deficits. The final row is restricted to the 3689 participants with any deficit and estimates the association per 0.10-unit higher FVI within that range. Abbreviations: CI, confidence interval; FVI, Functional Vulnerability Index; HR: hazard ratio.

### 3.6 Proportional hazards and time-specific estimates

Diagnostics indicated departure from proportional hazards for FVI (*p* < 0.001), with a survey-weighted FVI-by-log-time interaction HR of 0.65 (95% CI: 0.62–0.67), indicating an association that attenuated over follow-up (Table S7). The fully adjusted HR per 0.10-unit was therefore interpreted as an average over follow-up, and time-specific models were estimated. The association was strongest in the first 2 years (HR: 1.28; 95% CI: 1.22–1.35), and remained positive and significant from 2 to 5 years (HR: 1.18; 95% CI: 1.13–1.24) and beyond 5 years (HR: 1.12; 95% CI: 1.07–1.18).

### 3.7 Absolute five-year mortality

Weighted 5-year mortality increased from 3.2% (95% CI: 2.5%–3.9%) among adults with no deficit to 6.3% with 1 deficit, 15.9% with 2, and 23.0% (95% CI: 19.8%–26.1%) with 3 or more (Table S8). These percentages are survey-weighted absolute (unadjusted) 5-year mortality risks. The adjusted comparisons that follow are risk ratios from survey-weighted modified Poisson models. In fully adjusted modified Poisson models, the corresponding adjusted risk ratios reached 2.95 (95% CI: 1.99–4.35) for 2 deficits and 3.31 (95% CI: 2.49–4.41) for 3 or more, with a continuous estimate of 1.17 (95% CI: 1.13–1.21) per 0.10-unit.

### 3.8 Functional vulnerability vs. poverty and insurance

In a model with poverty and insurance but not FVI, poverty was associated with mortality; after FVI was added, the poverty association was no longer present (fully adjusted HR: 0.96; 95% CI: 0.76–1.21), and current uninsurance was not independently associated with mortality (HR: 1.12; 95% CI: 0.81–1.54) (Table S9). Established predictors behaved as expected, including age (HR per year, 1.07), female sex (HR: 0.60), current smoking (HR: 2.02), diabetes (HR: 1.38), and any cancer history (HR: 1.34).

### 3.9 Liver subgroup

Weighted mortality was higher among adults with chronic liver disease than among those with hepatitis only (21.5 and 25.2 *vs.* 13.5 deaths per 1000 person-years for chronic liver condition only and for both conditions, respectively), and mean FVI followed the same ordering (Table S10). In the primary model, chronic liver condition only (HR: 1.49; 95% CI: 1.22–1.81) and both conditions (HR: 1.51; 95% CI: 1.23–1.85) carried higher mortality than hepatitis only. The FVI-mortality association did not differ across liver subgroups (overall interaction *p* = 0.20).

### 3.10 Sensitivity, multiple imputation, and subgroups

Findings were stable across analyses (Table S11). Excluded participants differed only modestly from those included (Table S12), and multiple imputation reproduced the primary estimate (HR per 0.10-unit, 1.20; 95% CI: 1.17–1.23). Landmark analyses excluding deaths within 1 and 2 years yielded HRs of 1.17 (95% CI: 1.14–1.21) and 1.16 (95% CI: 1.12–1.20), indicating the association was not an artifact of imminent deaths. Estimates were unchanged by alternative FVI constructions, nonmissing-item thresholds, flexible age adjustment, leave-one-item-out indices (Table S13), and the chronic-liver-only and expanded cohort definitions (range of HRs: 1.16–1.20). Associations were consistent across age, sex, and race and ethnicity, with no evidence of interaction.

## 4 Discussion

Among community-dwelling US adults reporting a history of hepatitis or a chronic liver condition, a survey-based functional index showed a strong, graded association with all-cause mortality. The association was held after adjustment for demographics, poverty and insurance, health behaviors, cardio-metabolic comorbidity, and liver subgroup. The association also rose stepwise from a single deficit to three or more and translated into large differences in absolute 5-year risk, from roughly 1 in 30 among adults with no deficit to nearly 1 in 4 among those with the heaviest burden. These results extend hepatology frailty research from the transplant clinic and the cirrhosis ward to a nationally representative outpatient population that is rarely tested for frailty.

The association was non proportional, strongest in the first 2 years and attenuating thereafter. Part of that early excess likely reflects reverse causation, in which baseline deficits mark sub-clinical advanced disease in adults who die soon afterward. Two features argue against that being the whole story. First, the association persisted beyond 5 years. Second, analyses that removed deaths within the first 1 and 2 years left the estimate essentially unchanged. Functional vulnerability therefore appears to capture durable differences in physiologic reserve rather than only the shadow of impending death.^13^^,^^15^^,^^19^

Several pathways plausibly link functional vulnerability to death in liver disease. Cirrhosis and advanced fibrosis drive muscle depletion through impaired protein synthesis, hyperammonemia, increased myostatin signaling, endotoxemia, and chronic inflammation, and the resulting sarcopenia and malnutrition are themselves strong predictors of mortality.^30^^,^^55^^,^^56^ Functional limitation is the visible expression of that depleted reserve. Vulnerability also operates through behavior and access. For example, adults who struggle with mobility and daily tasks face transportation insecurity, social isolation, and weaker support, each tied to worse outcomes in liver disease.^57^, ^58^, ^59^ A survey-based functional measure may integrate these biological and social pathways into a single, clinically legible signal.

The contrast with conventional social markers is instructive. Poverty was associated with mortality until FVI entered the model, after which the association disappeared, while current uninsurance was not independently associated with death. This pattern should not be read as evidence that income or coverage are unimportant. A more careful interpretation is that functional vulnerability lies on the causal pathway between social adversity and death, so that adjusting for it removes part of the total effect of poverty rather than refuting it.^11^^,^^12^ Function may serve as a downstream, embodied summary of cumulative disadvantage, multimorbidity, and limited access that single social indicators measure only coarsely.

Characterization of the index supports its use for population-level description while clarifying what it is not. Internal consistency was high, and a single component dominated the tetrachoric structure; yet, we treat the FVI as a formative deficit-accumulation measure rather than a reflective psychometric scale, consistent with how frailty indices are constructed and interpreted.^15^^,^^16^^,^^60^^,^^61^ The graded, monotonic relationship with age, comorbidity, poverty, and liver subgroup, and the stepwise mortality gradient, are the properties expected of a valid vulnerability measure. The FVI is not a substitute for the LFI, a performance-based bedside tool for cirrhosis and transplant evaluation,^27^^,^^28^ rather, the two answer different questions, and the present findings suggest that meaningful functional risk is visible far earlier and more broadly than specialty cohorts alone would imply.

Because deficit accumulation predicts mortality across general and disease-specific populations,^18^^,^^19^ the approach is likely to generalize beyond liver disease. Adults with diabetes, hypertension, or chronic obstructive pulmonary disease, in whom frailty is also common, would plausibly show similar gradients, and brief functional assessment has been advanced as a practical strategy across chronic disease.^62^ The liver-specific contribution here is to show that the association is present, strong, and graded in a community population defined by hepatitis or chronic liver disease, and that it is not explained by the social and clinical factors usually invoked.

The implications for practice and surveillance are direct. A patient who reports poor health, difficulty with mobility, dependence on routine tasks, or reduced work capacity is at materially higher risk, whether or not that patient is insured or below the poverty threshold, and that risk is detectable with a handful of questions already collected in many settings. We deliberately stop short of proposing a screening cutoff, because the data identify a gradient rather than a validated threshold. The practical message is that any reported deficit warrants attention and that the highest-burden group carries roughly three times the risk. For health systems, the findings support pairing etiology-directed care with nutritional support, medication review, rehabilitation, and social-work referral. For surveillance, given the rising population burden of liver-related death,^63^ functional vulnerability deserves to be tracked as part of that burden rather than confined to transplant populations.

This study has limitations. Liver disease status was self-reported, and the public-use file lacks laboratory values, fibrosis stage, and markers of decompensation, so etiology and severity could not be characterized and misclassification is possible; non-differential misclassification of the cohort-defining condition chiefly affects who is studied and the labeling of the population rather than the internal validity of the FVI-mortality association within the cohort. Only all-cause mortality was available, because the public-use linked mortality files restrict and perturb cause-of-death detail,^43^ the conclusions therefore concern overall prognosis rather than liver-specific death. Follow-up time was approximated from survey year and death year and quarter, though landmark analyses that depend on follow-up timing were concordant. Complete-case modeling could introduce selection bias, but multiple imputation reproduced the estimate. The FVI is a survey-derived measure and was not validated against the LFI, residual confounding cannot be excluded, and the findings should not be generalized to hospitalized or transplant populations. Future work should collect objective, performance-based measures such as the LFI alongside the FVI in single-center clinical cohorts, enabling a direct head-to-head comparison that would benchmark this self-reported tool against an established frailty instrument.

## 5 Conclusions

Among community-dwelling US adults with a history of hepatitis or chronic liver disease, a survey-based functional phenotype was strongly associated with all-cause mortality, capturing prognostic information not explained by poverty or insurance status. Functional vulnerability may be a practical population-level marker of risk and a bridge between hepatology frailty and community liver disease epidemiology.

## CRediT authorship contribution statement

**Chukwuemeka E. Ogbu:** Writing – review & editing, Writing – original draft, Visualization, Validation, Software, Project administration, Methodology, Investigation, Formal analysis, Conceptualization. **Ifeanyi Momodu:** Writing – review & editing, Writing – original draft, Visualization, Validation, Supervision, Methodology. **Philip N. Okafor:** Writing – review & editing, Writing – original draft, Validation, Supervision, Methodology, Formal analysis.

## Informed consent

Not applicable. This study used deidentified, publicly available data and involved no direct participant contact or collection of identifiable patient information.

## Ethics statement

This study used deidentified, publicly available National Health Interview Survey data linked to public-use mortality files. Because the analysis involved secondary use of publicly available, deidentified data with no direct participant contact, institutional review board approval was not required.

## Organ donation

Not applicable.

## Animal treatment

Not applicable.

## Data availability statement

The data used in this study are publicly available from IPUMS NHIS and the National Center for Health Statistics Public-Use Linked Mortality Files. IPUMS NHIS data can be accessed through the IPUMS NHIS website, subject to its standard registration and data-use requirements. The National Health Interview Survey Public-Use Linked Mortality Files are available through the National Center for Health Statistics. The analytic code used for this study may be made available by the corresponding author upon reasonable request.

## Declaration of generative AI and AI-assisted technologies in the writing process

During the preparation of this manuscript, the authors used ChatGPT (OpenAI) and Grammarly for language editing, grammar, and formatting assistance only. These tools were not used to generate study data, perform statistical analyses, interpret the results, or make scientific conclusions. All AI-assisted text was reviewed and revised by the authors, who take full responsibility for the content and integrity of the manuscript.

## Funding

The authors received no specific funding for this work.

## Declaration of competing interests

The authors declare that they have no known competing financial interests or personal relationships that could have appeared to influence the work reported in this paper.
