# Supplementary material for: Functional vulnerability and all-cause mortality among community-dwelling United States adults with a history of hepatitis or chronic liver disease
Source: ILIVER. 2026 Aug 24;5(3):100259. doi: 10.1016/j.iliver.2026.100259 (PMC13571496; doi:10.1016/j.iliver.2026.100259)
Supplement: Supplementary file 1 [file mmc1.docx]

**Supplementary Material**

Supplementary tables S1–S13 are referenced in the main text.

**Table S1. Analytic sample counts**

| **Dataset** | **Unweighted No.** | **Deaths** | **Person-years** |
| --- | --- | --- | --- |
| FVI-eligible analytic sample | 10,910 | 1,418 | 70,518.0 |
| Primary complete-case model sample | 9,291 | 1,151 | 59,731.9 |

The FVI-eligible sample required at least 7 of 9 nonmissing FVI items. The primary complete-case model additionally required nonmissing covariates and liver subgroup.

**Table S2. FVI construct characterization summary**

| **Domain** | **Metric** | **Result** |
| --- | --- | --- |
| Distribution | Validation sample | *n* = 10,910; deaths = 1418; weighted mean FVI 0.152 (SE 0.003) |
| Distribution | Zero mass | FVI = 0 in 58.6%; FVI > 0 in 41.4% |
| Internal consistency | KR-20 / binary alpha | Weighted KR-20 0.901; unweighted alpha 0.907 (complete-item *n* = 6,954) |
| Item-total | Weighted corrected range | 0.437–0.841 |
| Pairwise items | Phi/Pearson range | 0.307–0.877 |
| Tetrachoric | Range | 0.687–0.991 |
| Structure | First component | Eigenvalue 7.787; 86.5% of variance |
| Structure | Second component | Eigenvalue 0.407; 4.5% of variance |
| Structure | Absolute PC1 loading range | 0.302–0.352 |

Note: These statistics characterize the survey-derived FVI and are interpreted descriptively because the FVI is a formative deficit-accumulation measure, not a reflective psychometric scale; they are not a validation against the Liver Frailty Index. Abbreviations: FVI, Functional Vulnerability Index; KR-20, Kuder-Richardson 20; PC1, first principal component; SE, standard error.

**Table S3. FVI item properties, missingness, item-total correlations, and first-component loadings**

| **FVI item** | **Nonmissing No.** | **Deficit No.** | **Weighted missing (%)** | **Weighted deficit prev (%)** | **Item-total r** | **PC1 loading** |
| --- | --- | --- | --- | --- | --- | --- |
| Fair/poor self-rated health | 10,906 | 2,929 | 0.0 | 24.6 | 0.592 | 0.302 |
| ADL help needed | 10,910 | 561 | 0.0 | 4.4 | 0.478 | 0.324 |
| IADL help needed | 10,909 | 1,124 | 0.0 | 8.4 | 0.586 | 0.332 |
| Work limitation | 10,908 | 2,992 | 0.0 | 23.9 | 0.649 | 0.326 |
| Walking difficulty | 9,633 | 2,007 | 11.1 | 17.5 | 0.841 | 0.352 |
| Climbing difficulty | 10,005 | 1,571 | 7.5 | 13.0 | 0.800 | 0.351 |
| Standing difficulty | 9,566 | 2,505 | 12.5 | 22.5 | 0.806 | 0.349 |
| Carrying difficulty | 10,026 | 1,277 | 7.1 | 10.8 | 0.729 | 0.346 |
| Grasping difficulty | 9,860 | 515 | 8.7 | 4.5 | 0.437 | 0.313 |

Note: Deficit prevalence is among participants nonmissing for the item. Corrected item-total correlations are weighted correlations between the item and the FVI proportion excluding that item. PC1 loadings are from the tetrachoric correlation matrix among participants with complete FVI items. Abbreviations: ADL, activity of daily living; FVI, Functional Vulnerability Index; IADL, instrumental activity of daily living; PC1, first principal component.

**Table S4. FVI component prevalence by observed deficit count**

| **FVI component** | **Overall** | **0** | **1** | **2** | **≥ 3** | ***p*** |
| --- | --- | --- | --- | --- | --- | --- |
| Fair/poor self-rated health | 2,344 (22.5) | 0 (0.0) | 525 (45.8) | 334 (58.7) | 1,485 (77.7) | < 0.001 |
| ADL help needed | 437 (3.8) | 0 (0.0) | 3 (0.1) | 8 (1.2) | 426 (21.1) | < 0.001 |
| IADL help needed | 851 (7.2) | 0 (0.0) | 7 (0.5) | 35 (5.6) | 809 (38.8) | < 0.001 |
| Work limitation | 2,438 (22.6) | 0 (0.0) | 424 (35.6) | 402 (69.3) | 1,612 (82.1) | < 0.001 |
| Walking difficulty | 1,548 (15.5) | 0 (0.0) | 31 (3.6) | 95 (21.9) | 1,422 (88.8) | < 0.001 |
| Climbing difficulty | 1,220 (11.4) | 0 (0.0) | 19 (1.4) | 33 (6.6) | 1,168 (73.1) | < 0.001 |
| Standing difficulty | 1,952 (20.3) | 0 (0.0) | 122 (13.0) | 179 (44.2) | 1,651 (92.5) | < 0.001 |
| Carrying difficulty | 985 (9.6) | 0 (0.0) | 23 (2.1) | 41 (9.1) | 921 (61.5) | < 0.001 |
| Grasping difficulty | 412 (4.1) | 0 (0.0) | 17 (1.8) | 27 (5.3) | 368 (25.2) | < 0.001 |

Note: Values are unweighted No. (survey-weighted %) of participants with the deficit present, within each observed deficit-count category. Abbreviations: ADL, activity of daily living; IADL, instrumental activity of daily living.

**Table S5. Known-groups associations: weighted mean FVI across expected vulnerability groups**

| **Domain** | **Group** | **No.** | **Weighted (%)** | **Mean FVI (SE)** | ***p*** |
| --- | --- | --- | --- | --- | --- |
| Age group (years) | 18–44 | 2,364 | 28.8 | 0.056 (0.004) | < 0.001 |
|  | 45–64 | 4,341 | 47.8 | 0.163 (0.005) |  |
|  | ≥ 65 | 2,586 | 23.4 | 0.188 (0.007) |  |
| Poverty status | Not below poverty | 7,465 | 85.4 | 0.110 (0.003) | < 0.001 |
|  | Below poverty | 1,826 | 14.6 | 0.298 (0.010) |  |
| Insurance status | Insured | 8,146 | 87.7 | 0.145 (0.004) | < 0.001 |
|  | Uninsured | 1,145 | 12.3 | 0.088 (0.007) |  |
| Comorbidity count | 0 | 4,217 | 48.2 | 0.060 (0.003) | < 0.001 |
|  | 1 | 3,081 | 31.6 | 0.158 (0.006) |  |
|  | ≥ 2 | 1,993 | 20.1 | 0.291 (0.010) |  |
| Liver subgroup | Hepatitis only | 6,172 | 67.1 | 0.102 (0.003) | < 0.001 |
|  | Chronic liver only | 1,765 | 19.0 | 0.191 (0.008) |  |
|  | Both conditions | 1,354 | 13.9 | 0.237 (0.011) |  |

Note: *p* values are overall design-based tests for differences in mean FVI across groups within each domain. The lower mean FVI among uninsured participants reflects their younger, predominantly pre-Medicare composition. Abbreviations: FVI, Functional Vulnerability Index; SE, standard error.

**Table S6. Mortality gradient by observed FVI deficit count**

| **Deficit count** | **No.** | **Deaths** | **Weighted death, % (SE)** | **Rate/1000 PY** |
| --- | --- | --- | --- | --- |
| 0 deficits | 5,602 | 334 | 5.2 (0.4) | 7.7 |
| 1 deficits | 1,171 | 144 | 10.7 (1.1) | 16.5 |
| 2 deficits | 577 | 127 | 19.5 (1.9) | 32.0 |
| ≥ 3 deficits | 1,941 | 546 | 28.2 (1.4) | 50.0 |

Note: Weighted mortality rates are deaths per 1000 person-years. Abbreviations: FVI, Functional Vulnerability Index; PY, person-years; SE, standard error.

**Table S7. Proportional-hazards diagnostics and time-specific estimates**

| ***A. Proportional-hazards diagnostics*** | | | | | | | | |
| --- | --- | --- | --- | --- | --- | --- | --- | --- |
| **Diagnostic** | | **Result** | | | ***p*** | | **Interpretation** | |
| Schoenfeld test, FVI | | *χ*² = 13,870.7 (df, 1) | | | < 0.001 | | Time-varying association | |
| Global Schoenfeld test | | *χ*² = 67,235.0 (df, 35) | | | < 0.001 | | Global nonproportionality | |
| FVI × log follow-up time | | HR 0.65 (0.62–0.67) | | | < 0.001 | | Attenuation over follow-up | |
| ***B. Time-specific survey-weighted Cox models*** | | | | | | | | |
| **Interval** | **No. at risk** | | **Deaths** | **Person-years** | | **HR per 0.10 (95% CI)** | | ***p*** |
| 0–2 years | 9,291 | | 268 | 18,385.2 | | 1.28 (1.22–1.35) | | < 0.001 |
| > 2–5 years | 8,321 | | 467 | 21,979.6 | | 1.18 (1.13–1.24) | | < 0.001 |
| > 5 years | 5,432 | | 416 | 19,367.0 | | 1.12 (1.07–1.18) | | < 0.001 |

The primary HR is interpreted as an average across follow-up; panel B reports estimates within prespecified intervals. CI, confidence interval; FVI, Functional Vulnerability Index; HR: hazard ratio.

**Table S8. Weighted 5-year all-cause mortality by observed functional deficit count**

| **Deficit count** | **Weighted 5-y risk, % (95% CI)** | **Adjusted RR (95% CI)** | ***p*** |
| --- | --- | --- | --- |
| 0 deficits | 3.2 (2.5–3.9) | 1 [Reference] |  |
| 1 deficits | 6.3 (4.3–8.3) | 1.33 (0.89–1.99) | 0.157 |
| 2 deficits | 15.9 (11.3–20.6) | 2.95 (1.99–4.35) | < 0.001 |
| ≥ 3 deficits | 23.0 (19.8–26.1) | 3.31 (2.49–4.41) | < 0.001 |
| Continuous, per 0.10 higher FVI | — | 1.17 (1.13–1.21) | < 0.001 |

Note: Absolute risks are survey-weighted 5-year death risks among 2008–2014 participants. Adjusted RRs are from survey-weighted modified Poisson models with the fully adjusted covariate set. Abbreviations: CI, confidence interval; FVI, Functional Vulnerability Index; RR, risk ratio.

**Table S9. Full primary adjusted Cox model coefficients**

| **Covariate** | **HR (95% CI)** | ***p*** |
| --- | --- | --- |
| FVI, per 0.10 higher score | 1.19 (1.15–1.22) | < 0.001 |
| Age, per year | 1.07 (1.06–1.08) | < 0.001 |
| Female *vs.* male | 0.60 (0.50–0.71) | < 0.001 |
| Non-Hispanic Black *vs.* White | 0.91 (0.70–1.18) | 0.487 |
| Hispanic *vs.* White | 0.94 (0.73–1.22) | 0.648 |
| Non-Hispanic Other *vs.* White | 0.56 (0.39–0.81) | 0.002 |
| High school/GED *vs.* < high school | 1.15 (0.92–1.45) | 0.226 |
| Some college/AA *vs.* < high school | 0.96 (0.76–1.21) | 0.727 |
| Bachelor+ *vs.* < high school | 0.90 (0.68–1.18) | 0.431 |
| Below poverty *vs.* not | 0.96 (0.76–1.21) | 0.704 |
| Uninsured *vs.* insured | 1.12 (0.81–1.54) | 0.497 |
| Former smoker *vs.* never | 1.22 (0.99–1.51) | 0.061 |
| Current smoker *vs.* never | 2.02 (1.60–2.55) | < 0.001 |
| Former drinker *vs.* lifetime abstainer | 1.27 (1.00–1.61) | 0.048 |
| Current drinker *vs.* lifetime abstainer | 0.96 (0.74–1.25) | 0.772 |
| Body mass index, per kg/m² | 0.97 (0.95–0.99) | 0.004 |
| Diabetes *vs.* no | 1.38 (1.15–1.65) | < 0.001 |
| Hypertension *vs.* no | 1.14 (0.95–1.37) | 0.157 |
| Prior heart attack *vs.* no | 1.23 (0.98–1.54) | 0.080 |
| Any cancer history *vs.* no | 1.34 (1.12–1.61) | 0.001 |
| Chronic liver condition only *vs.* hepatitis only | 1.49 (1.22–1.81) | < 0.001 |
| Both conditions *vs.* hepatitis only | 1.51 (1.23–1.85) | < 0.001 |

Note: Outcome is all-cause mortality. Region and survey-year coefficients are omitted for brevity. Reference categories are indicated in each label. Abbreviations: CI, confidence interval; FVI, Functional Vulnerability Index; HR: hazard ratio.

**Table S10. Liver/hepatitis subgroup outcomes and effect modification**

***A. Outcomes by subgroup***

| **Subgroup** | **No.** | **Deaths** | **Rate/1000 PY** | **Mean FVI (SE)** | **Weighted (%)** |
| --- | --- | --- | --- | --- | --- |
| Hepatitis only | 6,172 | 633 | 13.5 | 0.102 (0.003) | 67.1 |
| Chronic liver condition only | 1,765 | 259 | 21.5 | 0.191 (0.008) | 19.0 |
| Both conditions | 1,354 | 259 | 25.2 | 0.237 (0.011) | 13.9 |

***B. FVI × subgroup interaction***

| **Interaction contrast** | **Interaction HR (95% CI)** | ***p*** |
| --- | --- | --- |
| FVI × chronic liver condition only (*vs.* hepatitis only) | 0.95 (0.89–1.01) | 0.075 |
| FVI × both conditions (vs hepatitis only) | 0.99 (0.94–1.05) | 0.722 |
| Overall FVI × subgroup interaction | — | 0.200 |

Note: Mortality rates are survey-weighted deaths per 1000 person-years. Interaction terms compare the FVI slope in each subgroup with the hepatitis-only reference; the overall interaction used a design-based Wald test. Abbreviations: CI, confidence interval; FVI, Functional Vulnerability Index; HR: hazard ratio; PY, person-years; SE, standard error.

**Table S11. Sensitivity analyses of the association between functional vulnerability and all-cause mortality**

| **Analysis** | **No.** | **Deaths** | **HR per 0.10 (95% CI)** | ***p*** |
| --- | --- | --- | --- | --- |
| Primary complete-case Cox model | 9,291 | 1,151 | 1.19 (1.15–1.22) | < 0.001 |
| Multiple imputation (20 imputations) | 10,854 | 1,409 | 1.20 (1.17–1.23) | < 0.001 |
| Landmark: exclude deaths ≤ 1 y | 9,212 | 1,072 | 1.17 (1.14–1.21) | < 0.001 |
| Landmark: exclude deaths ≤ 2 y | 8,321 | 883 | 1.16 (1.12–1.20) | < 0.001 |
| Flexible age adjustment (natural spline) | 9,291 | 1,151 | 1.19 (1.15–1.22) | < 0.001 |
| Require ≥ 8 of 9 items | 8,030 | 896 | 1.19 (1.15–1.23) | < 0.001 |
| Require 9 of 9 items | 5,991 | 531 | 1.20 (1.15–1.26) | < 0.001 |
| FVI excluding self-rated health | 9,292 | 1,151 | 1.17 (1.14–1.20) | < 0.001 |
| FVI excluding work limitation | 9,291 | 1,151 | 1.18 (1.15–1.22) | < 0.001 |
| FVI excluding self-rated health and work | 9,292 | 1,151 | 1.16 (1.13–1.20) | < 0.001 |
| “Do not do activity” counted as deficit | 9,513 | 1,228 | 1.19 (1.16–1.22) | < 0.001 |
| Any physical difficulty counted as deficit | 9,291 | 1,151 | 1.19 (1.15–1.22) | < 0.001 |
| Chronic liver condition only cohort | 3,138 | 522 | 1.17 (1.12–1.22) | < 0.001 |
| Expanded cohort (recent liver condition) | 10,090 | 1,242 | 1.19 (1.16–1.22) | < 0.001 |

Note: Estimates are HRs per 0.10-unit higher FVI from the fully adjusted model. Construction analyses rebuilt the FVI under each alternative definition. The multiple-imputation estimate was pooled across 20 imputations by the Rubin rules. Abbreviations: CI, confidence interval; FVI, Functional Vulnerability Index; HR: hazard ratio.

**Table S12. Missing data and comparison of included *vs.* excluded participants**

| ***A. Inclusion after FVI eligibility*** | | | | | | | | | | | |
| --- | --- | --- | --- | --- | --- | --- | --- | --- | --- | --- | --- |
| **Group** | | | | **No.** | | | **Deaths** | | | **Person-years** | |
| Included in primary complete-case model | | | | 9,291 | | | 1,151 | | | 59,731.9 | |
| Excluded after FVI eligibility (missing covariates/subgroup) | | | | 1,619 | | | 267 | | | 10,786.1 | |
| ***B. Weighted comparison*** | | | | | | | | | | | |
| **Group** | **Death (%)** | **Mean age (year)** | | | **Mean FVI** | **Poverty (%)** | | | **Uninsured (%)** | | **Mean BMI (Kg/m^2^)** |
| Included | 10.7 | 53.0 | | | 0.138 | 14.6 | | | 12.3 | | 28.0 |
| Excluded | 9.3 | 53.6 | | | 0.171 | 19.5 | | | 16.8 | | 27.6 |
| ***C. Missingness among FVI-eligible participants*** | | | | | | | | | | | |
| **Variable** | | | **No. assessed** | | | | | **Missing, No. (%)** | | | |
| Standing difficulty | | | 10,910 | | | | | 1,344 (12.3) | | | |
| Walking difficulty | | | 10,910 | | | | | 1,277 (11.7) | | | |
| Grasping difficulty | | | 10,910 | | | | | 1,050 (9.6) | | | |
| Climbing difficulty | | | 10,910 | | | | | 905 (8.3) | | | |
| Body mass index | | | 10,910 | | | | | 901 (8.3) | | | |
| Carrying difficulty | | | 10,910 | | | | | 884 (8.1) | | | |
| Below poverty threshold | | | 10,910 | | | | | 628 (5.8) | | | |
| Alcohol use | | | 10,910 | | | | | 84 (0.8) | | | |
| Liver/hepatitis subgroup | | | 10,910 | | | | | 56 (0.5) | | | |
| Education | | | 10,910 | | | | | 43 (0.4) | | | |
| Insurance status | | | 10,910 | | | | | 22 (0.2) | | | |
| Prior heart attack | | | 10,910 | | | | | 16 (0.1) | | | |
| Hypertension | | | 10,910 | | | | | 14 (0.1) | | | |
| Smoking status | | | 10,910 | | | | | 10 (0.1) | | | |
| Cancer history | | | 10,910 | | | | | 9 (0.1) | | | |
| Fair/poor self-rated health | | | 10,910 | | | | | 4 (0.0) | | | |
| Work limitation | | | 10,910 | | | | | 2 (0.0) | | | |
| IADL help needed | | | 10,910 | | | | | 1 (0.0) | | | |

Note: Missingness is unweighted among FVI-eligible participants; variables with no missing values are not shown. The multiple-imputation analysis in Supplementary Table 2 addresses this missingness. Abbreviations: FVI, Functional Vulnerability Index; BMI, body mass index; IADL, instrumental activity of daily living.

**Table S13. Leave-one-FVI-item-out sensitivity analyses**

| **Excluded FVI item** | **No.** | **Deaths** | **HR per 0.10 (95% CI)** | ***p*** |
| --- | --- | --- | --- | --- |
| Fair/poor self-rated health | 9,292 | 1,151 | 1.17 (1.14–1.20) | < 0.001 |
| ADL help needed | 9,291 | 1,151 | 1.17 (1.14–1.20) | < 0.001 |
| IADL help needed | 9,291 | 1,151 | 1.18 (1.14–1.21) | < 0.001 |
| Work limitation | 9,291 | 1,151 | 1.18 (1.15–1.22) | < 0.001 |
| Walking difficulty | 9,832 | 1,247 | 1.18 (1.15–1.22) | < 0.001 |
| Climbing difficulty | 9,812 | 1,248 | 1.18 (1.15–1.22) | < 0.001 |
| Standing difficulty | 9,738 | 1,231 | 1.18 (1.15–1.22) | < 0.001 |
| Carrying difficulty | 9,691 | 1,224 | 1.17 (1.14–1.21) | < 0.001 |
| Grasping difficulty | 9,613 | 1,210 | 1.18 (1.15–1.21) | < 0.001 |

Note: Each row rebuilds the FVI after excluding the listed component and refits the fully adjusted model.

Abbreviations: CI, confidence interval; ADL, activity of daily living; FVI, Functional Vulnerability Index; HR: hazard ratio; IADL, instrumental activity of daily living.
